# Stress kinases, endoplasmic reticulum stress, and Alzheimer’s disease related markers in peripheral blood mononuclear cells from subjects with increased body weight

**DOI:** 10.1038/srep30890

**Published:** 2016-08-02

**Authors:** Ting Lei, Lugang Yu, Liqiang Qin, Baohui Xu, Lingmei Zhou, Jinbo Cheng, Hui Zhou, Xing Pang, Zhongxiao Wan

**Affiliations:** 1 Suzhou Industrial Park center Disease Control & Prevention, 58 Suqian Road, Suzhou, 215123, P.R. China; 2 Department of Nutrition and Food Hygiene, School of Public Health, Soochow University, 199 Renai Road, Suzhou, 215123, P.R. China; 3 Jiangsu Key Laboratory of Preventive and Translational Medicine for Geriatric Disease, Soochow University, 199 Renai Road, Suzhou, 215123, P.R. China

## Abstract

We aimed to characterize endoplasmic reticulum stress, inflammation, and Alzheimer’s disease (AD) related markers in peripheral blood mononuclear cells (PBMCs) from males with varied BMI; and to explore whether high glucose and fatty acids (FFAs) might be critical factors for inducing metabolic alterations in PBMCs under obese condition. Approximately 45 middle-aged men were enrolled with varied BMI. At the protein expression level, compared to the lean, the phosphorylation of AMPK, and p-Akt at serine 473 were significantly reduced from the overweight (OW) and/or obese (OB); while the protein expression of p-JNK, cleaved caspase 3, CHOP and p-eIF2α were elevated from the OW and/or OB. At the mRNA expression level, ER stress markers (i.e. GRP78, CHOP and XBP-1), inflammatory markers (i.e.TLR2, TLR4 and CCR2) and AD markers (i.e. APP, PS1 and PS2) were significantly higher in PBMCs from OB compared to lean. In cultured PBMCs, high glucose and FFAs induced GRP78, CHOP and XBP-1 mRNA, and high glucose also induced APP, PS1 and PS2 mRNA. In conclusion, altered markers including AMPK, ER stress and AD related makers under obese condition could be easily obtained from PBMCs. These markers might provide new mechanistic links between obesity and other metabolic complications including AD.

Worldwide obesity has become an epidemic^1^ and great progress has been made in understanding the etiology of obesity over the past two decades^2^,^3^. However, there is no long term effective strategy in the prevention and treatment of obesity, highlighting the importance of further exploring the complex biology of obesity.

Peripheral blood mononuclear cells (PBMCs) are usually defined as any blood cell with a round nucleus, which mainly include lymphocytes and monocytes^4^. PBMCs are one of the main antigen presenting cells found in the circulation, and PBMCs have been reported to play critical roles in immune response, metabolism, and communication with other cells. PBMCs are easily accessible and existing evidence also suggest a close association between PBMCs and obesity^5^,^6^, as well as type 2 diabetes (T2DM)^7^ and metabolic syndrome^8^. For example, mRNA expression of pro-inflammatory cytokines in PBMCs was associated with body fat distribution in normal weight young adults^6^. Collectively, PBMCs may represent a suitable model for exploring cellular aspects of obesity biology.

AMP-activated protein kinase (AMPK) is an energy sensing protein kinase and a master regulator of metabolic homeostasis, which regulates key enzymes involved in the metabolism of glucose and fatty acids^9^. Mitogen-activated protein kinases (MAPKs), including c-Jun N-terminal kinase (JNK), extracellular-signal-regulated kinase 1/2 (ERK1/2), and p38 MAPKs, are serine-threonine kinases that mediate diverse intracellular signalling pathways^10^. Abnormal ERK1/2 and p38 MAPKs activities have been reported in PBMCs from T2DM subjects, suggesting that high glucose and/or metabolic abnormalities may activate stress kinases in PBMCs^7^. However, there is no report whether AMPK and stress kinases activities in PBMCs will be altered between lean, overweight and obese subjects and the potential link between metabolic stress and altered MAPKs signaling in PBMCs is unclear. To elucidate the above issues will help us to understand the sequential alterations of AMPK and stress kinases activities in the pathogenesis of obesity, this will provide theoretical evidence for the prevention and treatment of obesity.

Endoplasmic reticulum (ER) is a critical cytoplasmic organelle that coordinates the synthesis, folding, and trafficking of proteins. Under stress conditions, unfolded proteins accumulate in the ER, this condition has been defined as ER stress^11^; in the meantime, an adaptive response known as the unfolded protein response (UPR) will be activated^11^. If the stress is too severe, the UPR may also induce cellular apoptosis via several mechanisms including the activation of p53 and cleaved caspase3^11^; as well as the activation of the JNK pathway, which is also associated with the development of insulin resistance^12^. ER stress is also closely associated with inflammation^13^. Consequently, ER stress has been recognized as a key mechanism involved in obesity related pathologies^11^. It remains unknown what might be critical factors for elevated ER stress markers under obese condition.

Obesity has been identified as a significant risk factor for increased risk of Alzheimer’s disease (AD)^14^. Amyloid precursor protein (APP), presenilin-1 (PS1) and PS2 are key proteins involved in the pathogenesis of AD^15^. Evidence suggests that APP, PS1 and PS2 are expressed in PBMCs^16^. Thus, it could be of interest to explore whether APP, PS1 and PS2 would be altered in PBMCs under obese condition and what might be the critical factors for inducing APP, PS1 and PS2 alteration.

Collectively, the aim of the present study is 1) to determine whether there are differences for stress kinases related signaling pathways, ER stress, inflammation and AD related markers (i.e. APP, PS1 and PS2) in PBMCs from Chinese middle-aged men with varied body mass index (BMI); and 2) whether high glucose and fatty acids (FFAs) might be critical factors for affecting ER stress and AD markers under obese condition.

## Materials and Methods

### Study Subjects

From October to December 2014, we enrolled 45 middle-aged men (age 45–60 years old) with no history of cardiovascular disease or T2DMs from Suzhou Industrial Park area, Suzhou, China. We recruited 15 subjects each for the lean (LN), overweight (OW) and obese (OB) group, respectively. According to the working group on obesity in China^17^, lean was defined as 18.5 ≤ BMI ≤23.9 kg/m^2^; OW was defined as 24.0 ≤ BMI ≤27.9 kg/m^2^; and OB was defined as BMI ≥28 kg/m^2^. The selection of sample size was mainly based on published findings from other research groups who have reported altered markers under obese condition with similar sample size^18^. The present study was conducted according to the guidelines laid down in the Declaration of Helsinki, and all procedures involving human subjects were approved by the Human Research and Ethical Committee of the Soochow University and all participants provided signed informed consent.

### Biological sampling, anthropometric and blood pressure measurement

Subjects reported to the laboratory after an overnight fast at 8 a.m. Blood samples (5 mL) were obtained by venipuncture from an antecubital vein. Blood was centrifuged at 1500 g for 10 mins at 4 °C and plasma immediately frozen at −80 °C for subsequent batch analyses. An additional 10 mL blood was collected into sodium heparin tubes for isolation of PBMCs (see below). Waist circumference (WC) was measured at the end of several consecutive natural breadths, at the level parallel to the floor, midpoint between the top of the iliac crest and the lower margin of the last palpable rib in midaxillary line. Hip circumference was measured at a level parallel to floor, at the largest circumference of buttocks. WHR was calculated by dividing WC (in cm) by hip circumference (cm). The height, body weight, systolic blood pressure (SBP), and diastolic blood pressure (DBP) of the subjects were measured by trained research assistants following standardized procedures using calibrated equipment.

### Materials

Ficoll Paque Plus (cat#17-1440-03) was from GE Healthcare (NY, USA). Glucose (cat#7528), fatty acid-free endotoxin-free bovine serum albumin (BSA; cat# A8806); and sodium salts of palmitate, linoleate, and oleate were from Sigma-Aldrich (MO, USA). Molecular weight marker (cat#1610373) and nitrocellulose membranes (cat#162-0115) for SDS-PAGE were from Bio-Rad (CA, USA). Immobilon western chemiluminescent HRP substrate (cat#WBKLS0100) was purchased from Millipore (MA, USA). Antibodies against phosphorylation (p)-AMPK&AMPK, p-ACC&ACC, p-ERK1/2&ERK1/2, p-p53, cleaved caspase 3, p-Akt at serine 473, p-p38&p38, p-JNK, 78 kDa glucose-regulated protein (GRP78), C/EBP homologous protein (CHOP), APP and beta-actin were from Cell Signaling (MA, USA). Antibodies against PS1 and PS2 were from Santa Cruz Biotechnology (CA, USA). The phosphorylation of protein kinase R (PKR)-like endoplasmic reticulum kinase (P-PERK) and the phosphorylation of the eukaryotic initiation factor 2α subunit (p-eIF2α) were from ImmunoWay Biotechnology Company (DE, USA). Human insulin ELISA kit (cat#EZHIASF-14K) was from Millipore (MA, USA). Universal RNA Extraction Kit, PrimeScript RT Master Mix kit and Premix Ex TaqTM (Probe qPCR) were from Takara Bio (Shiga, Japan). Taqman Gene Expression Assays for human GRP78 (Hs00607129_gh), CHOP (Hs00358796_g1), XBP-1 (Hs00231936_m1), TLR2 (Hs01872448_s1), TLR4 (Hs00152939_m1), CCR2 (Hs00704702_s1), APP (Hs00169098_m1), PS1 (Hs00997789_m1), PS2 (Hs01577197_m1), and eukaryotic 18S rRNA (4352930E) were from Applied Biosystems (CA, USA). All other chemicals were purchased from Sigma-Aldrich (MO, USA).

### PBMCs isolation

PBMCs were isolated by gradient density centrifugation of peripheral blood using Ficoll Paque Plus as described previously by our laboratory^19^. Briefly, two sets of 5 mL of blood was layered onto 5 mL of Ficoll Paque Plus in a sterile 15 mL tube and was centrifuged for 15 min at 800 g and at 20 °C. The layer of PBMCs was recovered and washed three times with sterile PBS for 10 min at 250 g at room temperature. Isolated PBMCs from these two tubes were then stored at −80 °C until further protein and mRNA expression analysis by western blotting and real-time RT-PCR, respectively.

### PBMCs *ex vivo* treatment

One week after the above PBMC isolation, 12 subjects from the lean group returned to the laboratory after an overnight fast at 8a.m. and PBMCs were isolated as described above. PBMCs (1 × 10^6^ of the target cells *per* well) were cultured in normal glucose (5 mM) RPMI 1640 media containing 10% fetal bovine serum. To mimic hyperglycemia and excess lipid exposure condition, PBMCs were treated with vehicle or high glucose (HG, 25 mM/L) or a mixture of FFAs (palmitate: linoleate: oleate, 2:2:1 ratio, 0.25 mM/L) complexed to FA-free low-endotoxin BSA for 24 hr. Thereafter, cells were collected for the mRNA measurement of ER stress and AD related markers. We chose these concentrations of glucose and FFAs mixture is based on published findings from others^20^ and our research group^19^. All *ex vivo* PBMCs culture experiment were run in duplicate.

### Plasma lipid profiles, glucose and insulin measurement

Triacylglycerol (TG), HDL-cholesterol, LDL-cholesterol, total cholesterol and glucose were measured on an automatic analyser (Hitachi7600, Tokyo, Japan). Plasma insulin was measured by ELISA following the manufacturer’s protocol with absorbance read on a microplate reader. All samples were run in duplicate. The coefficient of variation for duplicate samples in our lab is <10%. The homeostasis model assessment of insulin resistance (HOMA-IR) was calculated using the following equation: HOMA-IR = fasting insulin (FIns, μIU/ml) × fasting blood glucose (FBG, mmol/L)/22.5.

### Western blotting

Proteins from one set of PBMCs (isolated via above technique) were extracted. The phosphorylation and/or total protein expression of GRP78, CHOP, PERK, eIF2α, JNK, ERK1/2, p38MAPK, Akt, AMPK, ACC, p53 and cleaved caspase3 were determined by Western blotting following the methods published by our laboratory previously^21^. The loading volume of samples for the measurement of p53 and caspase3 is 30 μL, which is equal to 30 μg protein/well; the loading protein quantity is 20 μg/well for all the other proteins. Signals were visualized using Immobilon western chemiluminescent HRP substrate and bands were quantified by densitometry. All phosphorylated proteins were expressed relative to their respective total proteins or beta actin (if we couldn’t get the total protein). Beta actin was used as an internal control.

### Real-time RT-PCR

RNA was isolated from the other set of PBMCs using an RNeasy kit and 1 μg of RNA was used for the synthesis of complementary DNA using PrimeScript RT Master Mix kit. Real time PCR was performed using a 7500 Real-Time PCR system (Applied Biosystem). Results were normalized to the mRNA expression of 18S. Relative differences in gene expression between groups were determined using the 2^−∆∆CT^ method^22^. The amplification efficiencies of the gene of interest and the housekeeping gene were equivalent.

### Statistical analysis

All data are presented as mean ± SEM. Statistical analyses were performed using SPSS 20.0 (SPSS Inc., IL, USA). Data were analyzed for normality and homogeneity before statistical test. Comparisons between groups were evaluated via one-way ANOVA followed with Turkey’s post hoc analysis. Statistical significance was set at *p* < 0.05.

## Results

### Anthropometric parameter and lipid profiles

As illustrated in Table 1, subjects enrolled in the lean, OW and OB group were age matched. As expected, body weight, BMI, waist circumference (WC), hip circumference (HC), waist-hip ratio (WHR), SBP, as well as plasma TG, cholesterol, LDL-C and insulin were significantly higher in the OW and OB group compared to the lean group. Meanwhile, the HOMA-IR from OW group was significantly higher compared to the lean group. Plasma glucose and NEFAs were also significantly higher in the OB group compared to the lean group.

### Stress kinases signaling pathways from PBMCs

**Figure 1:**
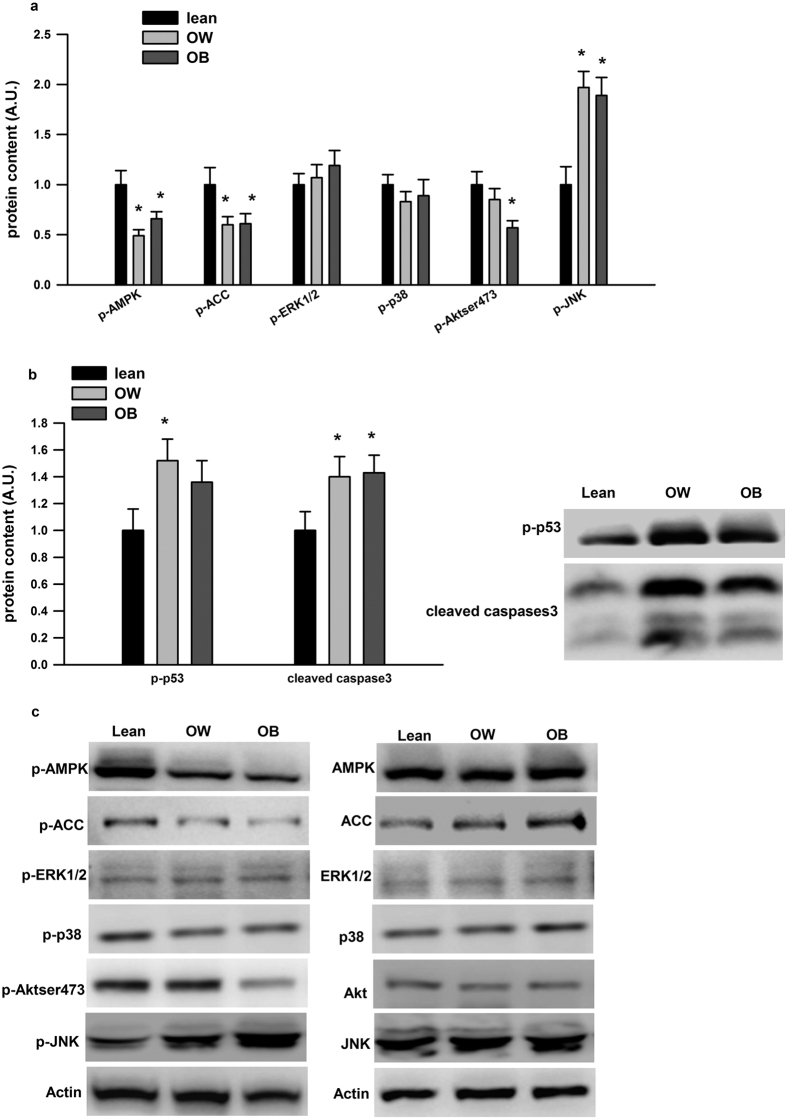
Stress kinases signaling pathways and apoptotic markers in PBMCs from subjects with varied BMI. (**a**) The phosphorylation of AMPK, ACC, ERK1/2, p38, Aktser473 and JNK and (**b**) p-p53 and cleaved caspase 3 in PBMCs from subjects were shown. Western blot images are given below the quantified data in (**b,c**). Data are presented as means + SEM for 15 subjects per group. *p < 0.05 versus lean group.

As shown in Fig. 1a, we first measured the phosphorylation of AMPK (p-AMPK), which is the active form of the enzyme^23^ and its downstream p-ACC. There were significant reductions in p-AMPK (50% and 34% reduction in the OW and OB group, respectively) and its downstream p-ACC (40% reduction in both groups) in PBMCs in comparison with the lean group; while p-JNK (about 2 fold increase in both OW and OB group) was significantly induced in PBMCs in comparison with the lean group. The phosphorylation of Akt at serine 473 was also decreased by 40% in PBMCs from the OB subjects compared to the lean subjects. There was no difference for the p-ERK1/2 and p-p38 between groups. Cleaved caspase 3 was also significantly increased by 40% in PBMCs from the OW and OB group compared to the lean group and p-p53 was also significantly increased by 50% from the OW group compared to the lean group (Fig. 1b).

### ER stress markers in PBMCs *in vivo* and *ex vivo*

**Figure 2:**
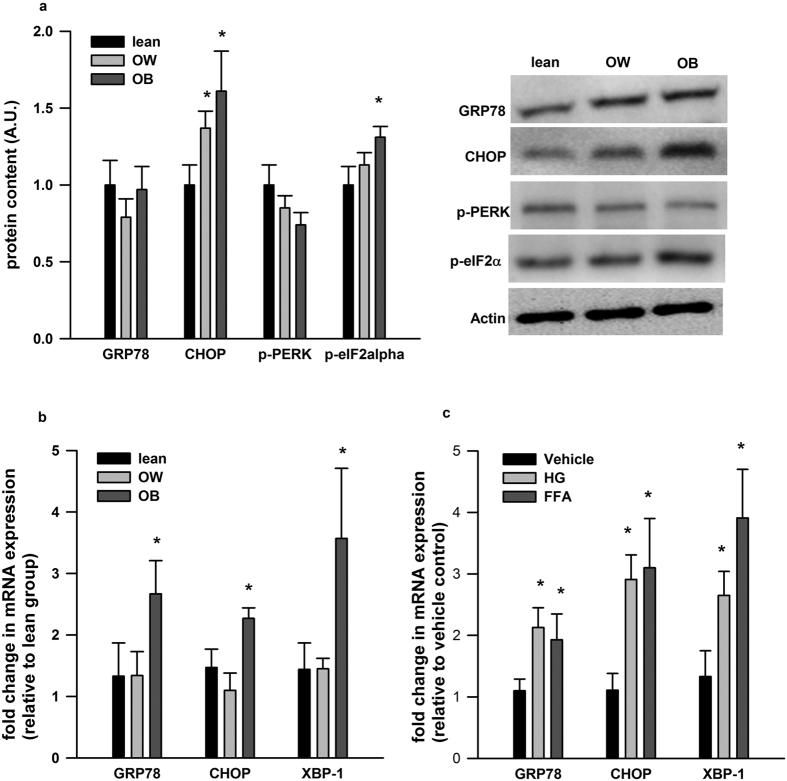
ER stress markers in PBMCs *in vivo* and *ex vivo*. (**a**) The protein expression of ER stress markers (i.e. GRP78, CHOP, p-PERK and p-eIF2α) and (**b**) the mRNA expression of GRP78, CHOP and XBP-1 in PBMCs from subjects were shown. (**c**) The mRNA expression of GRP78, CHOP and XBP-1 in cultured PBMCs incubated with high glucose (HG) and free fatty acids (FFA) were presented. Western blot images are given at the right of the quantified data in A. The mRNA data is normalized to 18S and expressed as fold differences compared to the lean group. Data are presented as means + SEM for 15 subjects *per* group in (**a,b**), and 12 subjects *per* group in (**c**). *p < 0.05 versus lean group in A&B. *p < 0.05 versus vehicle control in (**c**).

The protein expression of CHOP was significantly higher in PBMCs from the OW and OB group (about 40% and 60% increase, respectively) with respect to the lean group. The protein expression of p-eIF2α was also elevated in the OB subjects (about 30% increase) compared to the lean subjects (Fig. 2a). Similarly, there were approximately 2.5, 2 and 3.5 fold increase in the mRNA expression of GRP78, CHOP and XBP-1, respectively (Fig. 2b). Incubation of isolated PBMCs *ex vivo* from lean subjects with HG and FFAs mixture also significantly induced GRP78, CHOP and XBP-1 mRNA expression (Fig. 2c).

### AD related markers in PBMCs *in vivo* and *ex vivo*

**Figure 3:**
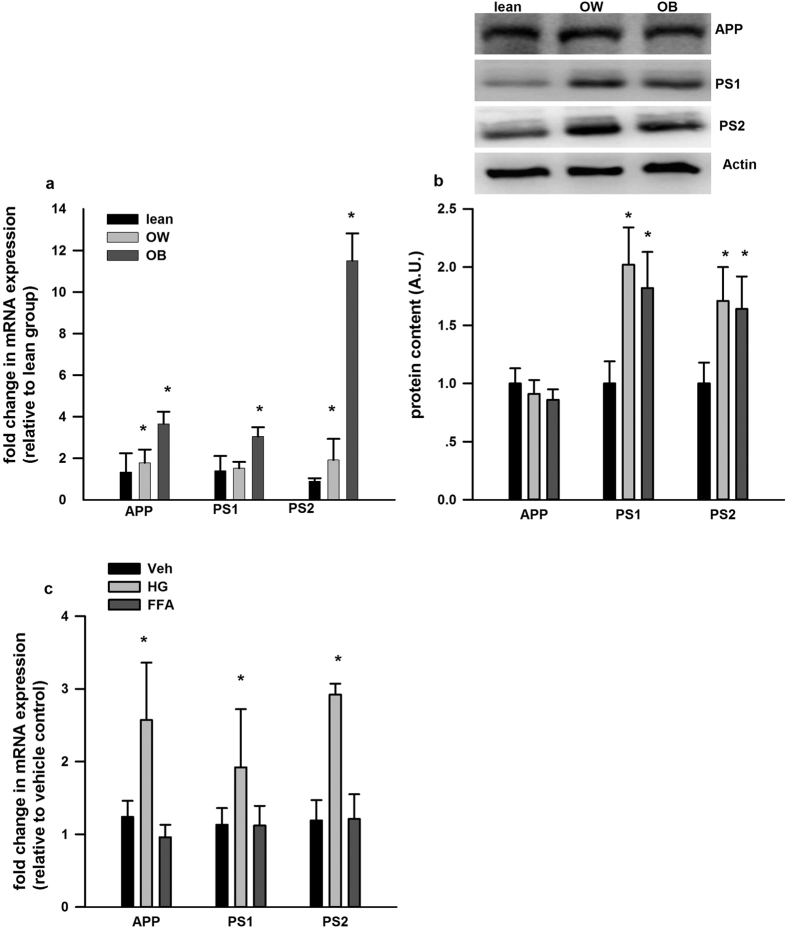
Gene expression of APP, PS1 and PS2 in PBMCs *in vivo* and *ex vivo*. (**a**) The mRNA (**b**) and protein expression of APP, PS1 and PS2 in PBMCs from different subjects were presented. (**c**) The mRNA expression of APP, PS1 and PS2 in cultured PBMCs incubated with HG and FFA were shown. Western blot images are given at the top of the quantified data in (**b**). The mRNA data is normalized to 18S and expressed as fold differences compared to the lean group. Data are presented as means + SEM for 15 subjects per group in (**a,b**), and 12 subjects in (**c**). *p < 0.05 versus lean group in A&B. *p < 0.05 versus vehicle control in (**c**).

There was significant increase in the mRNA expression of APP (1.8 and 3.7 fold, respectively) and PS2 (2 and 11 fold, respectively) in PBMCs from the OW and OB group with respect to the lean group. The mRNA expression of PS1 was also elevated (3 fold) in PBMCs from the OB group with respect to the lean group (Fig. 3a). We also measured the protein expression of APP, PS1 and PS2 in PBMCs via western blotting. As shown in Fig. 3b, compared to lean group, the protein expression of PS1 and PS2 were significantly elevated from OW and OB group, although no difference was observed for APP protein expression. When isolated PBMCs from lean subjects were treated with HG, there was a significant induction of APP, PS1 and PS2 mRNA expression, while FFAs mixture had no effect on APP, PS1 and PS2 mRNA expression (Fig. 3c).

### Inflammatory markers in PBMCs *in vivo*

**Figure 4:**
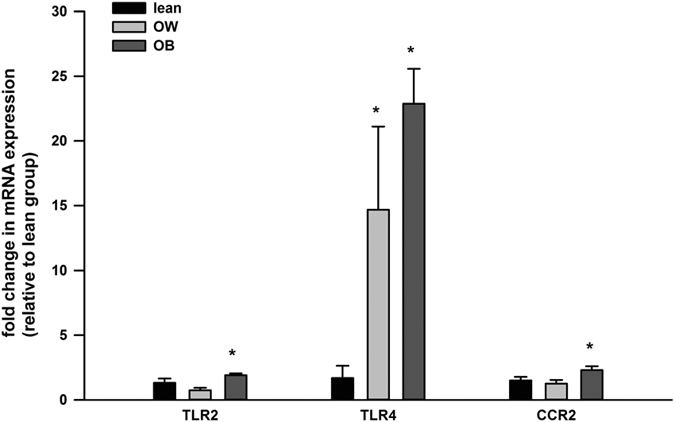
Gene expression of TLR2, TLR4 and CCR2 in PBMCs. The mRNA expression of TLR2, TLR4 and CCR2 in PBMCs from different subjects were shown. The mRNA data is normalized to 18S and expressed as fold differences compared to the lean group. Data are presented as means + SEM for 15 subjects per group. *p < 0.05 versus lean group.

The mRNA expression of TLR4 was significantly increased (15 and 23 fold, respectively) in PBMCs from the OW and OB group with respect to the lean group. The mRNA expression of TLR2 and CCR2 were also elevated (~2 fold) in PBMCs from the OB group with respect to the lean group (Fig. 4).

## Discussion

Our present study systematically demonstrated that in Chinese male adults the metabolic alterations in the transition from lean to obesity were accompanied by increased expression of stress kinases, as well as significant increase in the mRNA expression of ER stress markers (i.e. GRP78, CHOP and XBP-1), inflammatory markers (i.e.TLR2, TLR4, and CCR2) and AD markers (i.e. APP, PS1 and PS2). Our *ex vivo* experiment also suggested that elevated glucose and FFAs under overweight and obese conditions might be critical factors for inducing these metabolic alterations.

AMPK is a master regulator of metabolic homeostasis and AMPK activation is reduced in macrophage-infiltrated visceral adipose tissue of obese and insulin-resistant patients^24^. Our study is the very first to report that AMPK activity is reduced in PBMCs from both overweight and obese subjects. An increased basal activity of ERK1/2, p38MAPK and JNKs has been reported in adipocytes from patients with T2DM^25^. Previous studies also showed abnormal ERK and p38MAPK activities in monocytes of patients with T2DM^7^. However, in our present study there is no difference for stress kinases including p-ERK1/2 and p-p38 in PBMCs among lean, overweight and obese subjects. This could suggest that reduction in AMPK activity might be an earlier altered marker than p-ERK1/2 and p38 in PBMCs under obese condition. Meanwhile, we also observed a reduction in the phosphorylation of Akt in PBMCs from obese subjects coinciding with an elevation in circulating insulin levels. Akt phosphorylation states are usually considered as markers for insulin stimulation status within a tissue^26^. Findings from our study suggested that reduced p-Akt ser473 in PBMCs could reflect an impaired insulin stimulation status in obesity.

ER stress is considered as a critical contributor to obesity-related pathologies^11^. Work from Takamura and his colleagues reported that apoptosis related genes were up-regulated in PBMCs of T2DM patients^27^. Our study demonstrated that most of the ER stress markers were increased in PBMCs from obese subjects. This is in consistent with the findings by Degasperia *et al*.^18^ who reported activation of ER stress markers including GRP78, p-eIF2alpha and p-PERK in PBMCs from obese subjects. We further demonstrated that elevated glucose and FFAs under metabolic disorder conditions might be key factors for inducing ER stress markers in PBMCs. Previously, Alhusaini *et al*.^28^ reported that in human adipcoytes, high glucose and saturated fatty acids could also induce ER stress markers. In regards to the potential mechanisms, Kawasaki *et al*.^29^ reported that in adipocytes ER stress is induced by FFA-mediated reactive oxygen species (ROS) generation. Schisano *et al*.^30^ reported that in human umbilical vein endothelial cells, high glucose could induce ER stress via affecting mitochondrial fragmentation. It is likely that in our present study high glucose and fatty acids could also induce ER stress via affecting FFA mediated ROS and/or mitochondria fragmentation, however further studies are required to confirm these hypothesis. Meanwhile, we further observed that apoptotic markers were also elevated in PBMCs from overweight subjects. Collectively, our findings could suggest that ER stress response and apoptosis are pathological changes in PBMCs in obese compared to lean men; and that high glucose and FFAs might be key factors for inducing such alterations.

ER stress is also correlated with inflammation^13^. CCR2 belongs to a family of more than 50 proteins that play key roles in the recruitment of leukocytes at the site of inflammation^31^. Previously, Krinninger *et al*.^32^ reported that CCR2 expression was elevated in PBMCs from obese women, and our studies extended the findings to Chinese obese male subjects. Previously, Ahmad *et al*.^5^ have reported that TLR2 and TLR4 mRNA expression were increased in PBMCs from both overweight and obese subject, while we only observed elevated TLR4 but not TLR2 mRNA expression in PBMCs from Chinese overweight male participants. This could suggest that in Chinese middle-aged men, TLR4-mediated inflammatory signaling may be more related to obesity when compared to TLR2.

Our findings are the first to our knowledge to demonstrate that key molecules involved in the deposition of the amyloid plaques are elevated in PBMCs under both overweight and obese conditions. Of note, although APP mRNA expression was elevated while there was no difference for APP protein expression among groups. It has been well documented that APP undergoes extensive post-translational modifications such as glycosalation and phosphorylation^33^. The antibody against APP we utilized could only detect the total amount of APP, it is possible that some isoforms of APP might be altered under obese condition. Further studies are required to confirm this. We further demonstrated that elevated glucose but not FFAs might be critical factor for inducing APP, PS1 and PS2 mRNA expression in PBMCs. Yang *et al*.^34^ demonstrated that in both human neuroblastoma cells (i.e. SH-SY5Y) and non-neuronal HEK293 cells, high glucose could increase APP protein levels although with no effect on APP mRNA expression level. At this timepoint, we are uncertain how high glucose induced APP, PS1 and PS2 mRNA expression in PBMCs, clearly this is an interesting question requiring further exploration. Nevertheless, our present findings could suggest that the effects of glucose on AD pathogenesis is not limited to the brain, but also can systematically affect AD-related markers.

As discussed earlier, AMPK is an energy sensing protein kinase and a master regulator of metabolic homeostasis^9^. Mounting evidence also suggest that AMPK could counteract cellular abnormalities commonly existed in obesity such as inflammation, ER stress and oxidative stress^35^,^36^. Our present study also demonstrated that AMPK, inflammation, ER stress and AD related markers were altered in PBMCs under obese condition. Presumably, reduction in AMPK activity might be critical for all the other alterations observed in our study, while further studies are required to confirm this.

Our study is not without several limitations. First, we primarily focused on Chinese middle-aged men with a relatively small sample size, so the extrapolation of the results to other populations will be limited. Second, we could not track how long the subjects had been overweight or obese, it is likely the length of overweight and obese status might also be factors affecting the findings. Third, owing to technique and equipment limitation, we solely used BMI as a marker for lean, overweight and obesity classification, and we can’t determine the lean mass vs. fat mass.

## Conclusion

In conclusion, we demonstrated that 1) in Chinese middle-aged men, there are several molecular alterations in PBMCs between lean, overweight and obese status. This is mainly manifested by the activation of stress kinases, inflammation, ER stress, and apoptosis related markers; as well as induction of key molecules involved in the generation of Aβ; and 2) high glucose and FFAs might be critical factors for inducing ER stress markers in cultured PBMCs *ex vivo*, and high glucose also significantly induced APP, PS1 and PS2 mRNA expression in cultured PBMCs *ex vivo*. Further studies are required to explore whether the altered pathological alterations in PBMCs from subjects with increased adiposity might be key processes involved in the link between obesity and other chronic diseases such as T2DM and AD, as well as the potential roles of AMPK in these processes.

## Additional Information

**How to cite this article**: Lei, T. *et al*. Stress kinases, endoplasmic reticulum stress, and Alzheimer’s disease related markers in peripheral blood mononuclear cells from subjects with increased body weight. *Sci. Rep.* **6**, 30890; doi: 10.1038/srep30890 (2016).

## Figures and Tables

**Table 1:** Clinical and biochemical parameter of subjects from different groups.

Variables	Lean	Overweight	Obese
Age (years)	49.31 ± 0.88	48.88 ± 1.15	47.94 ± 1.44
BW (kg)	58.19 ± 1.26	73.21 ± 1.24*	83.72 ± 1.54*
BMI (kg/m^2^)	20.94 ± 0.34	25.78 ± 0.26*	28.81 ± 0.18*
WC (cm)	78.17 ± 1.57	91.25 ± 1.08*	97.06 ± 1.03*
HC (cm)	91.20 ± 1.12	99.15 ± 0.77*	103.25 ± 0.69*
WHR	0.86 ± 0.01	0.92 ± 0.01*	0.94 ± 0.01*
Glucose (mM)	4.93 ± 0.15	5.14 ± 0.12	5.35 ± 0.11*
Insulin (μM/mL)	2.55 ± 0.80	7.36 ± 1.38*	8.03 ± 1.81*
HOMA-IR	0.66 ± 0.07	1.31 ± 0.28*	1.03 ± 0.27
NEFAs (mM/L)	0.21 ± 0.03	0.20 ± 0.02	0.30 ± 0.04*
SBP (mmHg)	124.38 ± 2.78	130.50 ± 2.59*	135.63 ± 2.40*
DBP (mmHg)	80.60 ± 1.57	87.17 ± 1.47	85.25 ± 4.13
TG (mM/L)	0.91 ± 0.07	1.24 ± 0.12*	1.83 ± 0.36*
Cholesterol (mM/L)	4.03 ± 0.14	4.46 ± 0.14*	5.11 ± 0.21*
LDL-C (mM/L)	2.40 ± 0.13	2.79 ± 0.11*	3.20 ± 2.17*
HDL-C (mM/L)	1.17 ± 0.06	1.18 ± 0.05	1.13 ± 0.06

Data are presented as means + SEM for 15 subjects per group. *p < 0.05 versus lean group.
